# PTEN-AKT2 Regulates Mixed Lineage Liver Cancer Development and Sensitizes Cancer Cells to TGFβ Treatment

**DOI:** 10.21203/rs.3.rs-8697220/v1

**Published:** 2026-01-30

**Authors:** Qi Tang, Ielyzaveta Slarve, Jingyu Chen, Ni Zeng, Yiwei Gu, Lina He, Shunan Hu, Diala Alhousari, Zifei Xu, Brittney Hua, Guo Zhang, Phillip Nguyen, Mario Alba, Jian Xu, Baoan Ji, Shefali Chopra, Gary Kanel, Liyun Yuan, Bangyan L. Stiles

**Affiliations:** 1 Pharmacology and Pharmaceutical Sciences, Mann School of Pharmacy, University of Southern California, Los Angeles, CA 90033; 2 Cancer Biology, Mayo Clinic, Jacksonville, FL 32224; 3 Center for Craniofacial Molecular Biology, Herman Ostrow School of Dentistry, University of Southern California, Los Angeles, CA 90033; 4 Department of Medicine, Keck School of Medicine, University of Southern California, Los Angeles, CA 90033; 5 Department of Pathology, Keck School of Medicine, University of Southern California, Los Angeles, CA 90033

## Abstract

Primary liver cancers, including hepatocellular carcinoma (HCC) and cholangiocarcinoma (CCA), arise from the neoplastic transformation of hepatocytes and cholangiocytes, respectively. Loss or downregulation of PTEN, a tumor suppressor negatively regulating the PI3K/AKT pathway, is frequently observed in CCA and HCC. Notably, *PTEN* mutations are observed at nearly twice the frequency in combined CCA-HCC tumors than either HCC or CCA alone. Using lineage-specific liver-targeted PTEN-deficient mouse models, we demonstrate that PTEN loss drives cellular dedifferentiation and tumorigenesis, a process that is critically dependent on AKT2. Mechanistically, PTEN deficiency induces activation of NOTCH and upregulation of transcriptional factor SOX9, which plays a central role in tumor cell transformation. In parallel, PTEN loss increases SMAD4 expression and sensitizes the tumor cells to TGFβ signaling, with TGFβ treatment repressing SOX9 expression in tumor cells lacking PTEN. Together, our study defined a critical role for PTEN-AKT2 signaling in maintaining liver epithelial lineage fidelity and revealed how its disruption promotes the conversion of mature hepatocytes or cholangiocytes into liver cancer stem-like cells (LCSCs). Furthermore, we identify a PTEN-dependent crosstalk between NOTCH and TGFβ pathways that governs liver tumor development. Together, this work provides mechanistic insight into lineage plasticity in liver cancer with implications for pathway-directed therapy.

## Introduction

Hepatocellular carcinoma (HCC) and intrahepatic cholangiocarcinoma (iCCA) are the most common primary malignancies in the liver. Hyperproliferative cholangiocytes and their association with liver cancer stem–like cell (LCSC) markers confer an aggressive phenotype in iCCA and combined HCC–CCA (cHCC-CCA). Consistent with high rates of therapeutic resistance and relapse^1–3^, the median relative survival for mixed cHCC-CCA and iCCA is approximately 8 months and 7 months respectively (SEER*Stat database^4^). This mortality rate is projected to increase in the US, in contrast to the declining trends observed in most other major cancers^5^. Recent whole-genome and epigenetic analyses revealed substantial molecular heterogeneity within iCCA, with frequent alterations in *isocitrate dehydrogenase1 (IDH1)*, *BRCA1-associated protein1*, and *FGFR2* gene fusions associated with poor clinical outcomes^2,6^. Overexpression of ERBB family receptor tyrosine kinases, including EGFR and c-Met, similarly predicts adverse prognosis. Notably, aberrant activation of *FGFR2*, EGFR and c-Met drives tumor progression through downstream engagement of phosphoinositide 3-kinase (PI3K)/AKT and ERK^7,8^. Moreover, iCCAs harboring *IDH1* mutations also exhibit enhanced PI3K/AKT activity^9^, indicating convergence at this oncogenic pathway across distinct genetic subtypes.

PTEN (phosphatase and tensin homolog deleted on chromosome 10), a central tumor suppressor and negative regulator of the PI3K/AKT pathway^10^, is frequently downregulated in liver cancers with iCCA indications, with loss of expression reported in up to 70% of cases^11^. In murine models with liver-specific *Pten* deletion, we previously reported the development of mixed lineage tumors, underscoring the critical role of PTEN in cHCC-CCA development^12–14^. Here, we demonstrated that intact PTEN is necessary to maintain both cholangiocytes and hepatocytes in their differentiated states, whereas PTEN loss in either lineage is sufficient to drive cHCC-CCA development. Mechanistically, we identified AKT2 as the key downstream effector that promotes mixed lineage tumor formation, as it converges on SOX9 to sustain LCSC transformation, with opposing regulatory inputs from the NOTCH and TGFβ signaling pathways. Specifically, NOTCH signaling activates SOX9, whereas TGFβ suppresses SOX9 expression, and PTEN status determines the cellular responsiveness to TGFβ inhibition. Collectively, these findings establish AKT2 as a critical oncogenic driver in cHCC-CCA and reveal a PTEN-dependent regulatory axis in which TGFβ-mediated control of SOX9 governs LCSC transformation and tumor aggressiveness. Importantly, our data indicate that PTEN loss sensitizes tumor cells to TGFβ inhibition, highlighting potential therapeutic vulnerabilities in mixed-lineage liver cancers.

## Materials and Methods

### Patient Samples

All human studies were performed under protocols approved by the University of Southern California (USC) Institutional Review Board and conducted in accordance with the ethical principles of the Declarations of Helsinki and Istanbul. Formalin–fixed, paraffin-embedded tissue sections were obtained from the USC Tissue Bank under protocols HS-20–00168 (LY) and HS-16–00258 (BLS).

### Animals

The LiPten (*Pten*^LoxP/LoxP;^ Alb-Cre^+/−^) and ChoPten (*Pten*^loxP/loxP^*; R26R*^YFP^; SOX9-Cre^ERT^+Tamoxifen) mice were previously reported^13,15^. HepPten mice were generated via injection of AAV8-TBG-Cre to *Pten*^*loxP/loxP*^; *R26R*^*YFP*^ at 1 month of age. LiPtenA2 (*Pten*^LoxP/LoxP^; *Akt2*^LoxP/LoxP^; Alb-Cre^+/−^) mice were developed by crossing *Akt2*^*loxP/loxP*^ (LiA2) mice^16^ with the LiPten mice. Control mice were Cre^−/−^ mice from the respective cohorts. Male mice on C57BL/6J background were housed under standard conditions with ad libitum access to food and water and a 12-hour light/dark cycle. All animal procedures were approved by the Institutional Animal Care and Use Committee at the University of Southern California and conducted in accordance with institutional and national guidelines.

### Database Analysis

Median survival in patients with carcinoma of liver and intrahepatic bile duct or HCC or CCA was estimated using the Kaplan–Meier method in SEER*Stat with 95% confidence intervals obtained from the summary statistics^4^ (SEER 1975–2022; November 2024 Submission, accessed Nov 11, 2025). Genomic mutation data for liver and biliary tract cancers were obtained from cBioPortal^17^ (accessed June 21, 2025). TCGA and GTEx datasets were analyzed using GEPIA2 (http://gepia2.cancer-pku.cn/) to evaluate patient survival and differential gene expression between tumor and normal tissues in the Liver Hepatocellular Carcinoma and Intrahepatic Cholangiocarcinoma cohorts^18^. Expression profiling data analyzed in this study were reported to GEO (GSE70501)^19^. ChIP-seq and ATAC-seq data for HepG2 cells were downloaded from ENCODE^20^ and processed using standard IGV pipelines.

### Cell Culture

Huh7 (JCRB0403)^21^, PLC/PRF/5 (ATCC, #CRL-8024)^22^ and mouse immortalized cholangiocytes^23^ were cultured in DMEM (Corning Inc., #10–013-CV) supplemented with 10% FBS (Neuromics, #FBS007). Mouse immortalized liver cell lines (WT, *Pten*^*−/−*^, *Pten*^*−/−*^*; A2*^*−/−*^) were established from WT, LiPten, LiPtenA2 mouse livers and cultured as previously reported^24,25^. Cells were treated with DAPT (N-[N-(3,5-difluorophenacetyl)-L-alanyl]-S-phenylglycine t-butyl ester) (Cayman Chemical, #13197) or recombinant human TGFβ1 protein (Abcam, #ab50036) as indicated. SOX9 knockdown and NICD1 overexpression were achieved using siRNA- and plasmid-based approaches as described in the Supplementary Methods.

### Sphere and Colony Formation

Log-phase growing cells (25,000) were seeded in ultra-low attachment 24-well plates (Corning, #3473) in DMEM/F12 medium (Corning, #10–090-CV) and allowed to form spheres for up to 5 days. For serial passaging, primary spheres were dissociated into single cells to generate secondary and tertiary spheres using the same protocol. Spheres ≥100 μm in diameter were counted at the indicated time points.

For colony formation, log-phase cells (1250 per well) were suspended in 0.35% agar and overlaid onto a base agar layer (2 mL per well). Cultures were maintained at 37°C with 5% CO_2_ for 2–3 weeks, with fresh medium added every 3–4 days.

### Immunoblotting

Total protein was isolated from cultured cells or liver tissue homogenates using RIPA lysis buffer as previously described^26^. Samples were resolved by SDS-PAGE and transferred onto polyvinylidene fluoride membranes. Membranes were incubated with the primary antibodies (see Supplementary Methods). The HRP-conjugated secondary antibodies and ECL substrate were applied to visualize the protein bands.

### Immunofluorescence Staining

Paraffin-embedded tissue sections were deparaffinized, rehydrated, and subjected to antigen retrieval as described^27^. Primary antibodies (see Supplementary Methods) were incubated overnight at 4°C. Alexa Fluor-conjugated secondary antibodies (Thermo Fisher, 1:200) were applied for 1 hour at room temperature. Nuclei were counterstained with DAPI and imaged by Zeiss Axio Imager M1 fluorescence microscopy.

### RNA Analysis

Total RNA was extracted from cultured cells and mouse liver tissue using TRIzol reagent (Invitrogen, #15596018). Reverse transcription was performed with the M-MLV reverse transcriptase system (Promega, PR-M1705). Quantitative PCR (qPCR) was then conducted using the APExBIO qPCR kit (#K1070). Gene-specific primers are listed in Supplemental Methods.

### Statistical Analysis

Two-tailed Student’s t-test (2 samples) or ANOVA with Tukey posthoc test (>2 samples) were conducted for statistical analysis. * indicates p value < 0.05, ** indicates p value < 0.01, *** indicates p value < 0.001. Data represented as Mean ±SEM.

## Results

### Mutation of *PTEN* is associated with mixed tumor phenotype with iCCA indications and poor tumor staging

**Figure 1.**
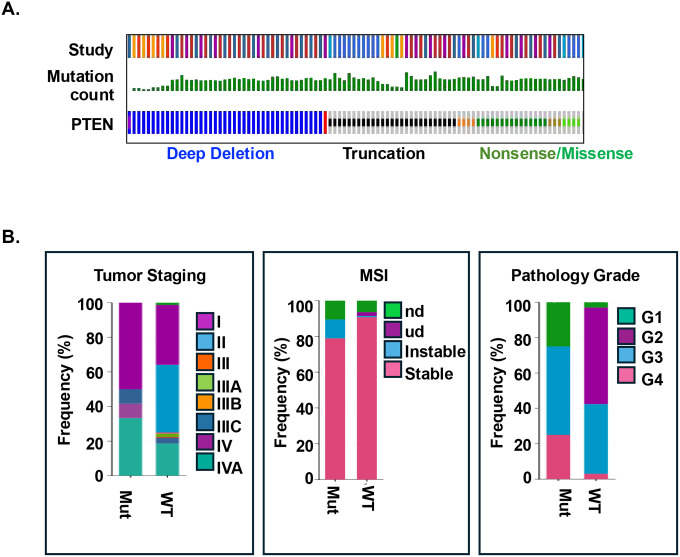
Mutation of *PTEN* is associated with mixed tumor phenotype with iCCA indications and poor tumor staging. **(A)** Mutation counts for *PTEN* in liver and biliary tract cancer samples accessed through cBioportal. Samples are from 30 studies including hepatobiliary cancer (84.2%), hepatocellular carcinoma (HCC, 6.2%), cholangiocarcinoma (CCA, 1.5%) and cancers of the biliary tract (0.4%) for a total of 5,519 patients and 5,648 samples. **(B)** Correlation analysis of *PTEN* mutation with tumor staging and histology scores.

The PTEN/PI3K signaling pathway has been considered a permissive cell growth signal that allows tumor development in the liver. We reported previously that loss of the tumor suppressor PTEN (LiPten mice) leads to the development of dual-lineage liver cancer after chronic steatosis and inflammation^14,19,28^. To understand the significance of PTEN-regulated signals in liver cancer, we performed mutational analysis of patient data available in cBioPortal^17^. Besides previous reported mutations of *p53* (32.4%), *CTNNB1* (14.5%), *ARID1A* (13.3%) and *TERT* (13.2%), mutations of genes within the PTEN-regulated pathway were also observed (*PIK3CA*,4%; *TSC2*,3.2%, and *PTEN*, 2.4%). Deep deletion, truncation and nonsense or missense mutations accounted for most of the mutations for *PTEN* (Fig. 1A). We further explored the mutations of *PTEN* in samples with iCCA indications. Among the 1,312 samples, 4% carried mutations of *PTEN* and 4.9% with *PIK3CA* mutations, ranking among the topmost frequently mutated genes. The mutation rate of *PTEN* was dramatically higher (7%) in samples that were clinically defined as both HCC and iCCA (190 HCC+iCCA; 49 iCCA+HCC), consistent with the development of dual lineage tumors observed in the LiPten livers^13,28^.

Using the American Joint Committee on Cancer TNM staging system, we observed that *PTEN* mutation was associated with higher tumor staging, particularly with tumors that presented distant metastasis (stage IV) and those with lymph node involvement (Stage IIIC) (Fig. 1B). *PTEN* mutation was also associated with the lowest grade tumors (stage I), whereas those with middle stages had a lower frequency of *PTEN* mutations. Thus, *PTEN* mutation may allow cells to gain transformation advantage that permits tumor initiation as well as additional metastasis potential. Consistently, more microsatellite instability (MSI) was observed in samples with *PTEN* mutation vs. those with wild-type *PTEN*. Histology grading of the samples indicates that *PTEN* mutation was associated with more undifferentiated or poorly differentiated tumors (Fig. 1B). Together, these patient data analyses suggest that PTEN may play a crucial role in maintaining hepatocytes and cholangiocytes in differentiated states to prevent tumor development.

### PTEN loss in the liver parenchyma leads to heterogeneous tumors

**Figure 2.**
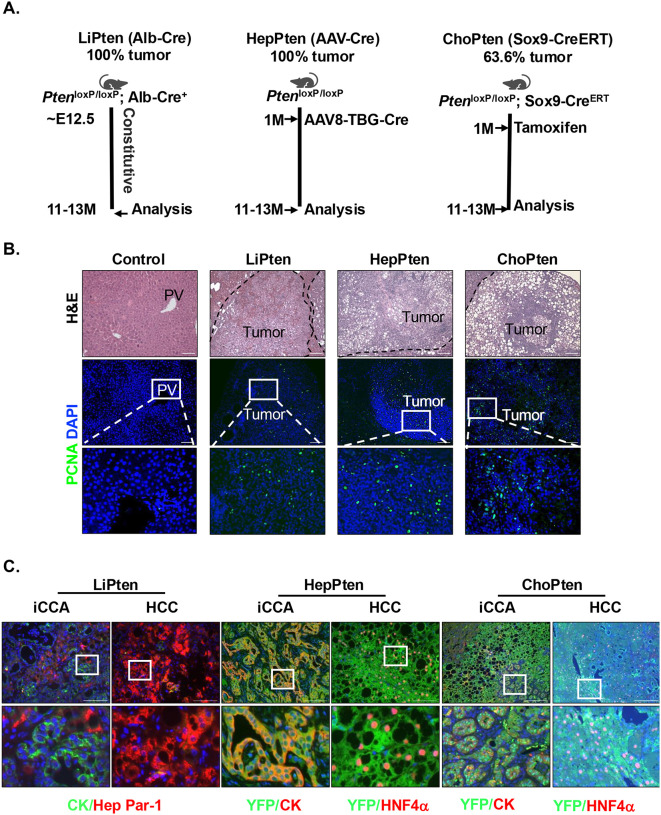
PTEN loss in liver parenchymal leads to heterogeneous tumors. **(A)** Schematics for generation of *Pten* deletion mouse models. LiPten is generated by crossing *Pten*^*loxP/loxP*^ mice with *Alb-Cre*^+^ mice where albumin expression starts in embryonic day E12.5 and cells targeted include hepatocytes and hepatoblasts. HepPten mice is generated by injection of AAV8-TGB-Cre virus into the *Pten*^*loxP/loxP*^*; YFP*^*lox-Stop-lox*^ mice at 1 month of age, which targets only mature hepatocytes. ChoPten mice are generated by tamoxifen injection at 1 month of age in *Pten*^*loxP/loxP*^*; YFP*^*lox-Stop-lox*^ *Sox9*^*CreERT*^ mice, which targets mature cholangiocytes. **(B)** Representative images of the 12-month mouse liver tissues from the respective mouse models (Top panels). Lower two panels, immunofluorescent staining of PCNA (green), co-stained with DAPI (blue) to show nuclei. **(C)** Immunofluorescent staining of HepPar-1 or HNF4a and CK to identify HCC and iCCA areas, respectively. The tissues are co-stained with DAPI (blue) to show nuclei. Scale bar 50 μm.

Here, we explored how PTEN and PI3K/AKT pathways regulate the development of the dual lineage tumors using mouse models carrying deletions of *Pten* and *Akt2*, the most abundant *Akt* isoforms expressed in the liver^29,30^. The LiPten mice develop dual lineage tumors when deletion of *Pten* is targeted to albumin-expressing cells^14,15^. Histologic examination by hematoxylin and eosin (H&E) staining revealed tumor regions characterized by the presence of both trabecular hepatocellular and glandular biliary-type malignant architectures (Fig. 2A). Staining for proliferating cell nuclear antigen (PCNA) demonstrated robust nuclear positivity within these tumor regions (Fig. 2B). To address whether the tumors originates from embryonic hepatoblasts, we developed two lineage tracing models that target *Pten* deletion to either mature hepatocytes or cholangiocytes using AAV8-TBG-Cre delivered to 1-month-old *Pten*^*loxP/loxP*^*; R26R*^*YFP*^ mice (HepPten) or tamoxifen-induced Cre expression in 1-month old *Pten*^*loxP/loxP*^*; R26R*^*YFP*^*; SOX9-Cre*^*ERT*^ mice (ChoPten)^13^ respectively (Fig. 2A&B and **supplemental Fig. 1**). All LiPten and HepPten mice developed tumors at 11–13 months of age with tumor incidence in the ChoPten mice being lower (63.6%) (Fig. 2A). Such reduced tumor incidence observed in ChoPten mice could result from less severe steatotic liver injury, since PTEN is still expressed in hepatocytes which prevents excessive lipid accumulation^13^. Similar to the LiPten mice that develop HCC (HepPar-1^+^) and iCCA (CK+), both HCC (HNF4a^+^) and iCCA (CK^+^) are observed in HepPten and ChoPten mice though with different penetrations (Fig. 2B&C). These findings suggest that PTEN loss indeed promotes the mixed lineage tumors regardless of the cell types that are targeted for *Pten* deletion. Lineage tracing analysis further demonstrated that both CK^+^ iCCA and HNF4a^+^ HCC tumors in these mice are positive for the lineage marker yellow fluorescent protein (YFP), indicating that the cells are derived from AAV8-TBG-Cre targeted hepatocytes and tamoxifen induces SOX9^+^ cells, respectively (Fig. 2C). Together, these genetic interrogations suggest that PTEN and the PTEN-regulated signals play critical roles in maintaining cellular plasticity of the liver cells.

### Loss of AKT2 rescues oncogenesis in livers lacking PTEN

**Figure 3.**
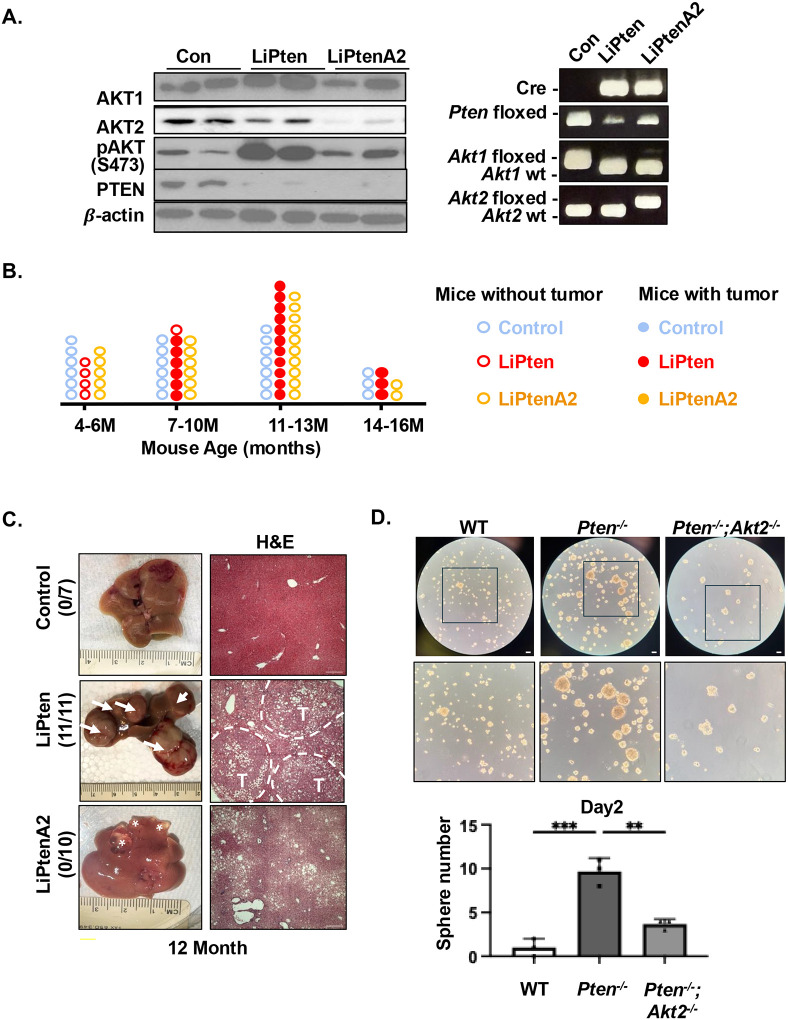
Loss of AKT2 rescues oncogenesis in livers lacking PTEN. **(A)** Genotyping and immunoblotting verified PTEN and AKT status in the respective mouse models. Left, liver tissue lysates analyzed with immunoblot; Right, tail DNA samples analyzed using PCR genotyping. Blots are cropped for clarity. **(B)** Tumor spectrum of WT, LiPten, and LiPtenA2 mice from 3–16 months. Each circle represents one mouse. Solid circles indicate tumor-bearing mice where open circles are mice without tumors. **(C)** Representative images of the 12-month old mouse livers from the respective genotype. Left, gross morphology. Right, H&E stained images. Arrows indicate tumor nodules, asterisks indicate cysts. T, tumor. Scale bar 100 μm. **(D)** Sphere formation was performed using immortalized liver cells established from WT, LiPten (Pten^−/−^) and LiPtenA2 (Pten^−/−^; AKT2^−/−^) mice. Shown in images (Top) are day 3 spheres. Bottom, quantification of the spheres at day 2. Scale bar 50 μm. n=4 * P<0.05. Experiment repeated 3 times.

We showed previously that germline loss of AKT2 inhibits tumor development in the LiPten livers and that this result is due to the metabolic effect of AKT2 loss on liver steatosis and associated injury^14,19,31^. To address whether AKT2 regulates the PTEN-controlled cholangiocyte-hepatocyte fate and associated oncogenesis, we deleted the liver *Akt2* in LiPten mice to develop mice lacking both AKT2 and PTEN in albumin-expressing cells (LiPtenA2, *Akt2*^*loxP/loxP*^; *Pten*^*loxP/loxP*^; Alb-Cre^+^) (Fig. 3A). Our data show that loss of AKT2 in the liver arrested the *Pten* deletion-induced tumorigenesis (Fig. 3B&C). At 12 months of age, only benign cysts were observed in the LiPtenA2 livers (Fig. 3B&C). These cysts do not progress to tumors until 15 months of age, indicating that AKT2 plays a critical role in the malignant transformation regulated by PTEN. To further explore this pro-tumorigenic role of AKT2, we performed sphere-forming assay with immortalized hepatocytes established from WT, LiPten and LiPtenA2 livers to assess their self-renewal and stemness properties in anchorage-independent, non-adherent 3D conditions. While WT hepatocytes rarely form spheres (>100μM), *Pten*^*−/−*^*; Akt2*^*−/−*^ hepatocytes form many fewer spheres compared to *Pten*^*−/−*^ hepatocytes. (Fig. 3D). Together, these data suggest that AKT2 and AKT2-regulated functions serve as critical regulators in malignant transformation in response to PTEN loss.

Consistent with a role of PTEN/PI3K/AKT signal in CCA development, the expression of all 4 *PIK3* genes was higher in iCCA vs. their respective non-tumor controls with *PIK3CA* and *PIK3CB* being significantly upregulated in iCCA but not HCC samples (**supplemental Fig. 2A**). A similar trend was observed for expressions of the AKT isoforms and their targets (**supplemental Fig. 2B**) with the expression of *AKT2* being significantly upregulated in iCCA but not HCC samples. Expression of *AKT3* is also significantly higher though *AKT3* expression is low in both normal and tumor liver tissues^29,30^. Furthermore, expression of *PTEN* negatively correlated with overall survival of iCCA but not HCC cohort (**supplemental Fig. 2C**). Together with the phenotype observed with LiPten and the LiPtenA2 mice (Fig. 2&3), these analyses suggest that the PTEN-regulated PI3K/AKT2 signaling may play a more critical role in iCCA and cHCC-CCA development than in HCC alone.

### NOTCH-SOX9 signal is induced in iCCA-HCC developed with PTEN loss

**Figure 4.**
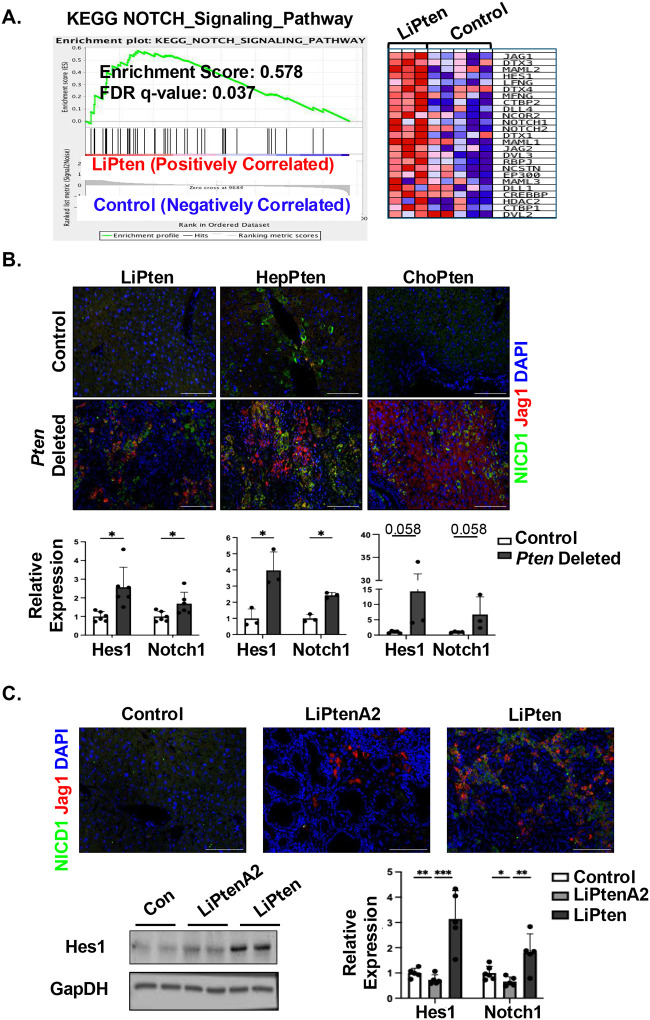
NOTCH-SOX9 signal is induced in iCCA-HCC developed with PTEN loss. **(A)** Gene Set Enrichment Analysis (GSEA) of KEGG Notch Signaling Pathway in 12-month-old LiPten vs control mice (GSE70501). Left, enrichment plot. Right, heatmap for genes that were differentially expressed in LiPten vs. control livers. **(B)** Top, immunofluorescent staining of NICD (Notch intracellular cleaved domain, green) and Jag1, a Notch ligand (Red) in the LiPten, HepPten and ChoPten livers. Tissues are co-stained with DAPI for nuclei (Blue). Bottom, qPCR analysis showing relative expression of Hes1 and Notch 1 in the LiPten (n=6, left), HepPten (n=3, middle) and ChoPten (n=3, right) livers. **(C)** Top, immunofluorescent staining of NICD (green) and Jag1 (Red) showing lack of signal in LiPtenA2 samples. Tissues are co-stained with DAPI for nuclei (Blue). Bottom, immunoblotting and qPCR analysis showing reduced Notch target Hes1 and Notch1 expression in LiPtenA2 vs. LiPten livers. Blots are cropped for clarity. n=5. Scale bar 50 μm. * p< 0.05, ** p< 0.01, *** p< 0.001.

Exploring how PTEN/AKT2-regulated signals promote iCCA and cHCC-CCA, our transcriptomic analysis (GSE70501) identified robust enrichment of the Notch signals in the LiPten livers compared to the controls. Receptors *Notch1 and 2*, ligands *Jagged1& 2* (*Jag1&2*), *Delta-like1&4* and Notch transcriptional target *Hes1* are among the top-enriched genes within this pathway analysis (Fig. 4A). NOTCH signal is critical for ductal development during embryogenesis and abnormal activation of NOTCH signal has been shown to induce the transformation of hepatocytes to malignant cholangiocytes and promote CCA development^32,33^. Similar to our observation for PIK3 and AKT isoforms (**supplemental Fig. 2**), expression of NOTCH signaling molecules is also upregulated in patient samples of iCCA and not HCC (**supplemental Fig. 3A**). Additionally, the expression of NOTCH signaling molecules, including *NOTCH1* and *JAGGED1*, correlated strongly with that of molecules within the PI3K/AKT signaling pathway (**supplemental Fig. 3B**).

We performed immunostaining of JAG1 and NICD, the cleaved and activated NOTCH intracellular domain, in the livers of the three tumor models (LiPten, HepPten and ChoPten) (Fig. 4B). Our data showed that JAG1 and NICD were robustly detected in tumors developed in these livers. Upregulated mRNA expression of *Notch 1* and its target gene *Hes1* supported this finding (Fig. 4B). In the LiPtenA2 livers, NICD is not detected and JAG1 was present only in the cystic ducts (Fig. 4C). Furthermore, loss of AKT2 significantly inhibited the expression of *Hes1* and *Notch1* induced by deletion of *Pten* (Fig. 4C). HES1 protein was also lower in LiPtenA2 livers than that observed in LiPten livers which is higher than controls. Thus, NOTCH activation may play a role in the iCCA and iCCA-HCC tumors driven by PTEN loss and activation of PI3K/AKT2.

**Figure 5.**
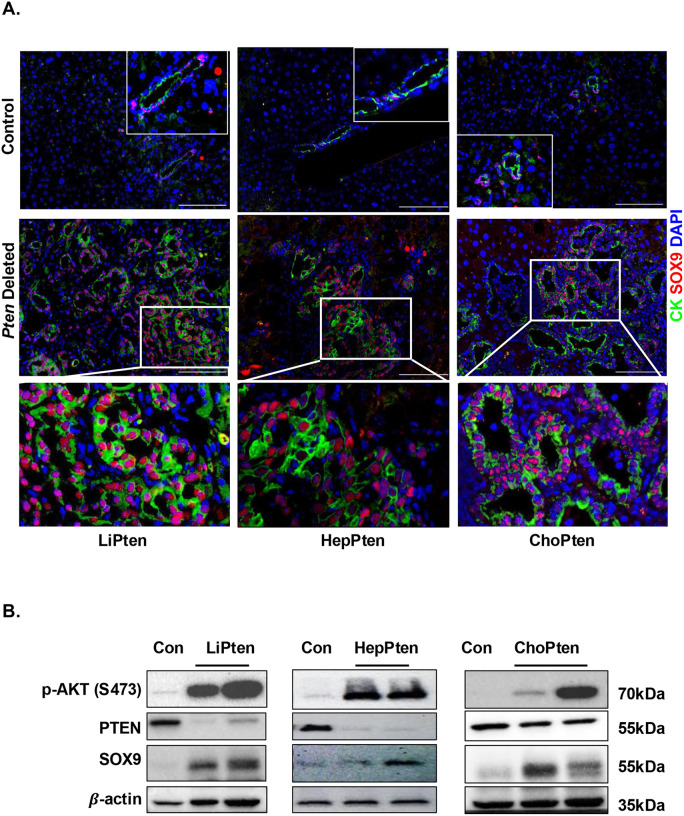

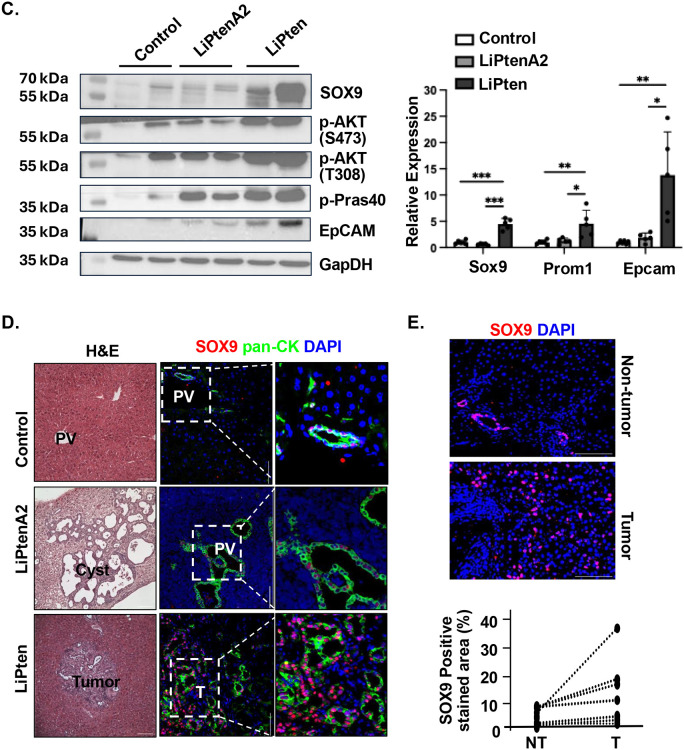
Induction of SOX9 by PTEN loss and its rescue by AKT2 loss. **(A)** Representative images of the 12-month mouse liver from LiPten (left), HepPten (middle) and ChoPten (right) mice stained with SOX9 (Red) and CK (Green). Tissues are co-stained with DAPI for nuclei (Blue). **(B)** Immunoblotting analysis of the 12-month mouse liver showing elevated SOX9 in LiPten, HepPten and ChoPten mouse livers. **(C)** Left, immunoblotting showing induction of SOX9 in LiPten and its inhibition in livers from LiPtenA2 mice. Blots are cropped for clarity. p-AKT (S473 and T308) shows AKT phosphorylation status that correlates with its activity as indicated by p-Pras40. EpCAM is also probed to assess LCSC marker expression. Right, qPCR analysis of the 12-month mouse liver showing induction of Sox9 and two other LCSC markers in the LiPten livers and its reduction in LiPtenA2 livers. n=5. * p< 0.05, ** p< 0.01, *** p< 0.001. **(D)** Representative H&E and immunofluorescent images showing induction and disorganized distribution of SOX9 (red) in LiPten livers and reduced expression in LiPtenA2 livers. Pan-CK (green) is stained to show cholangiocytes and ductal structures. DAPI (Blue) is stained to indicate nuclei. **(E)** Immunofluorescent staining of SOX9 (Red) protein expression in patient tumor samples vs. adjacent non-tumor tissues. Blue, DAPI. Scale bar 50 μm. Bottom, quantification of the staining. Dotted lines connect samples from the same patient.

Previous work has suggested that NOTCH cascade drives CCA development^32,33^ and that SOX9 may be involved in this effect^32,34^. Our co-expression analysis showed a strong correlation of *SOX9* with *NOTCH1* and *JAG1* in TCGA liver cancer samples (**supplemental Fig. 4A**). In tumors developed in all murine models– LiPten, ChoPten, and HepPten - SOX9 staining was detected, particularly in iCCA regions (Fig. 5A&B). Upregulation of pAKT and SOX9 was also observed when PTEN proteins were lost (HepPten and LiPten) or diminished (in ChoPten livers due to intact PTEN in untargeted hepatocytes) (Fig. 5B). Aligning with the lack of tumor development, SOX9 expression was significantly lower in the LiPtenA2 livers (Fig. 5C). Histological analysis showed that the macroscopic cysts developed in the LiPtenA2 livers were composed of dilated but well-organized ductal structures (Fig. 5D). Expression of SOX9 is localized in cholangiocytes within these organized ductal structures in the LiPtenA2 livers vs. the randomly scattered pattern observed in the LiPten livers (Fig. 5D).

In patient samples, immunostaining shows that SOX9 was highly expressed in tumor samples vs. adjacent tissues (Fig. 5E). In the TCGA data repository, SOX9 expression was more significantly induced in iCCA vs. HCC (**supplemental Fig. 4B**). Expression of SOX9 also negatively correlates with disease-free patient survival in iCCA (HR=1.6) but not HCC (HR=0.9) (**supplemental Fig. 4C**). Together, these data suggest that induction of SOX9 likely plays a role in the tumor transformation process regulated by PTEN/AKT2.

### NOTCH-SOX9 signal partially regulates tumor cell transformation controlled by PTEN-AKT2

**Figure 6.**
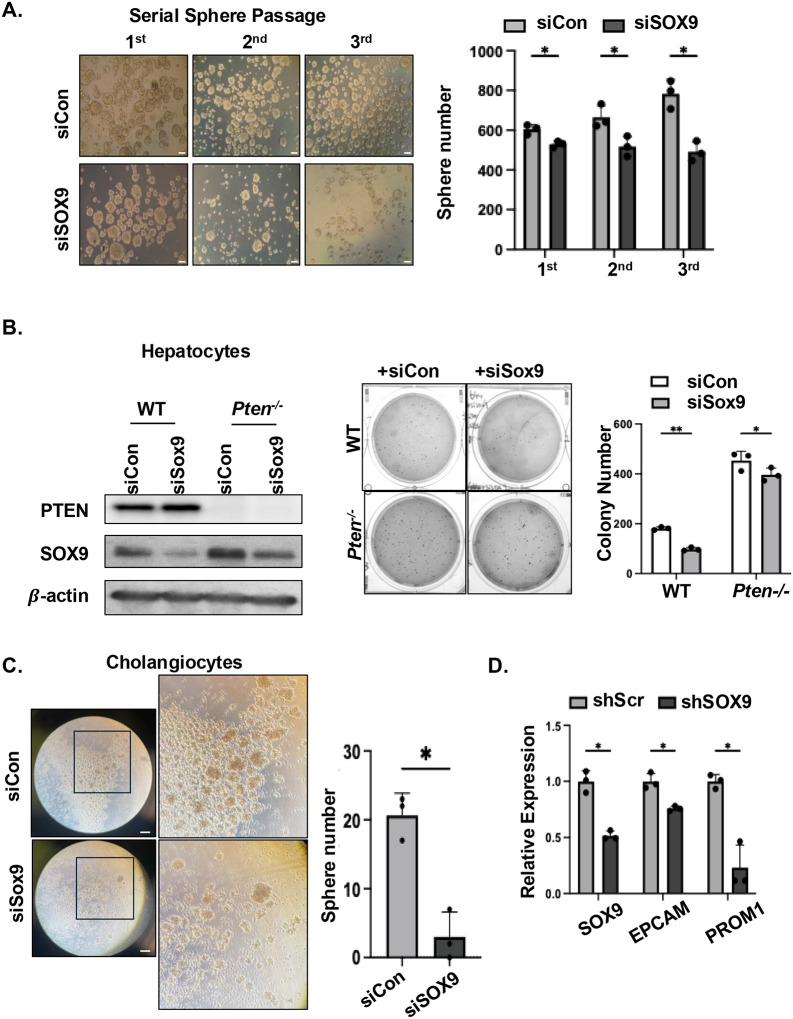
SOX9 regulates the transformation of LCSC. **(A)** Serial sphere formation was performed in Huh7 stem-like cells with the expression of *siSox9* or control siRNA. Spheres are broken up and passaged every 5 days and each passage counts as a series. Left, representative image of the spheres. Right, quantification of the spheres per 25,000 cells plated. **(B)** Colony formation assay performed using WT and *Pten*^*−/−*^ immortilized cells with or without *siSox9* transfection. Left, immunoblotting showing reduced levels of SOX9, blots are cropped for clarity; Middle, representative image of the colonies; Right, quantification of colonies. n=3. Experiment was performed twice. **(C)** Sphere formation assay performed using mouse immortalized cholangiocytes lacking PTEN with or without siSOX9 introduction (n=3). Left, representative image. Right, quantification of the spheres. **(D)** qPCR analysis showing down-regulation of stem cell markers with shSOX9 transfected in HepG2 cells. n=3. Scale bar 100 μm. * p< 0.05, ** p< 0.01.

Accumulating evidence suggests that SOX9 serves as a liver progenitor cell marker and that SOX9^+^ cells are capable of regenerating both hepatocytes and cholangiocytes after injury^35–37^. Supporting the role of SOX9 in sustaining LCSC state, expression of LCSC markers *EpCAM*^36^ and *CD133* (ref.^12^) strongly correlated with that of SOX9 in TCGA samples (**supplemental Fig. 5A**). Consistent with the regulation of SOX9 by PTEN-AKT2 signal, expressions of *Epcam* and *Prom1* are robustly induced in the LiPten livers while AKT2 loss attenuated their expression significantly (Fig. 5C). To test the potential of SOX9 in LCSC transformation, we knocked down *Sox9* and showed inhibiting Sox9 expression resulted in reduced sphere and colony formation ability using Huh7 (ref.^21^) and PLC/PRF/5 (ref.^22^) cells, which both exhibit LCSC characteristics (Fig. 6A and **supplemental Fig. 5B**). Thus, these data indicated that the presence of SOX9 indeed sustains a LCSC phenotype. In the *Pten*-deleted hepatocytes^24^ and cholangiocytes^23^, we also observed fewer spheres and colonies when *Sox9* was knocked down (Fig. 6B&C). Inhibiting SOX9 expression also reduced the expression of LCSC markers (Fig. 6D) such as PROM1 and EpCAM^12,36^. Together, these data suggest that PTEN loss permits the LCSC transformation, with induction of SOX9 playing a role in this process.

**Figure 7.**
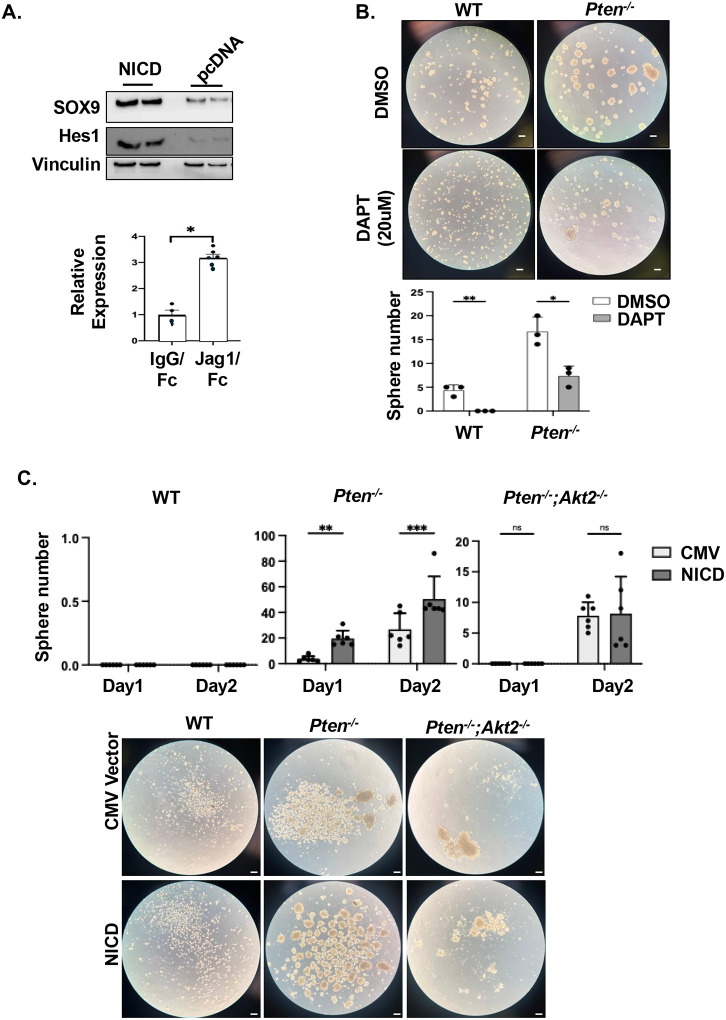
NOTCH-SOX9 signal partially regulates tumor cell transformation controlled by PTEN-AKT2. **(A)** Notch intracellular domain (NICD) was transfected to Huh7 cells. Expression of SOX9 and Hes1 was analyzed with Vinculin as a loading control. Blots are cropped for clarity. Bottom, Huh7 cells were exposed to Jag1 ligand- or IgG control-coated extracellular matrix and expression of SOX9 was quantified using qPCR. n=6. **(B)** Sphere formation of WT and *Pten*^*−/−*^ immortalized cells treated with or without DAPT. Top, representative images of the spheres. Bottom, quantification of the sphere numbers. n=3. Experiment repeated 3 times. **(C)** Sphere formation of WT, *Pten*^*−/−*^ and *Pten*^*−/−*^*; Akt2*^*−/−*^ immortalized cells with or without NICD transfection. Top, quantification of the sphere numbers. n=6. Bottom, representative images of the spheres. Scale bar 100 μm. * p< 0.05, ** p< 0.01, *** p< 0.001.

During ductal plate development, NOTCH signal is necessary to program the cholangiocyte fate partially by inducing the upregulation of SOX9. Gain-of-function experiments showed that ectopic expression of NICD in or exposing Huh7 cells to JAG1 ligand-coated extracellular matrix both induced the expression of SOX9 (Fig. 7A). Thus, NOTCH signal indeed positively regulates the expression of SOX9 in liver cancer cells. To address whether pharmacological inhibition of NOTCH can attenuate tumor cell transformation due to PTEN-AKT2 alteration, we performed sphere formation using *Pten*^*−/−*^ mouse liver tumor cells treated with or without DAPT, the γ-secretase inhibitor that blocks NOTCH Signaling^38^. As expected, the *Pten*^*−/−*^ cells formed larger and more spheres than the WT cells (Fig. 7B). DAPT treatment significantly reduced sphere formation in both cell lines with less pronounced differences observed for the *Pten*^*−/−*^ cells.

We next explored if introducing the NICD could rescue the inability of sphere formation due to AKT2 loss (Fig. 7C and **supplemental Fig. 6**). Introduction of NICD appeared to permit the WT cells to aggregate more though none reached the size threshold for spheres (>100μM) by day 3, the end of the experiment. Introduction of NICD further increased the number of spheres formed by the *Pten*^*−/−*^ cells. Surprisingly, the introduction of NICD was not able to alter the sphere size in the *Pten*^*−/−*^*; Akt2*^*−/−*^ cells. Of note, the *Pten*^*−/−*^*; Akt2*^*−/−*^ cells appeared to be sensitive to transfection stress, as most cells did not survive the culture conditions. Together, our data suggest that while inhibiting NOTCH activation blocks PTEN loss induced LCSC transformation, activating NOTCH itself is not sufficient to drive LCSC transformation when AKT2 signal is absent. Thus, NOTCH signal may only be partially responsible for the PTEN-AKT2-regulated LCSC transformation.

### PTEN loss sensitizes tumor cells to TGFβ regulated LCSC repression

To determine the signals regulated by PTEN/AKT2 that intersect with the NOTCH–SOX9 axis during tumor transformation, we examined the role of TGFβ signaling. Previous studies have shown that TGFβ induces Sox9 expression during ductal development^39–41^. However, in bipotent progenitor models including HepaRG and hepatoblast-like cells, TGF-β was reported to promote hepatocyte differentiation while suppressing biliary markers like Sox9 (ref. 39–41), indicating context-dependent effects. We showed previously that combining loss of PTEN and SMAD4 accelerates tumor development and shifts tumor identity toward iCCA^24^, implicating TGFβ signaling as a modifier of lineage fate downstream of PTEN loss.

**Figure 8.**
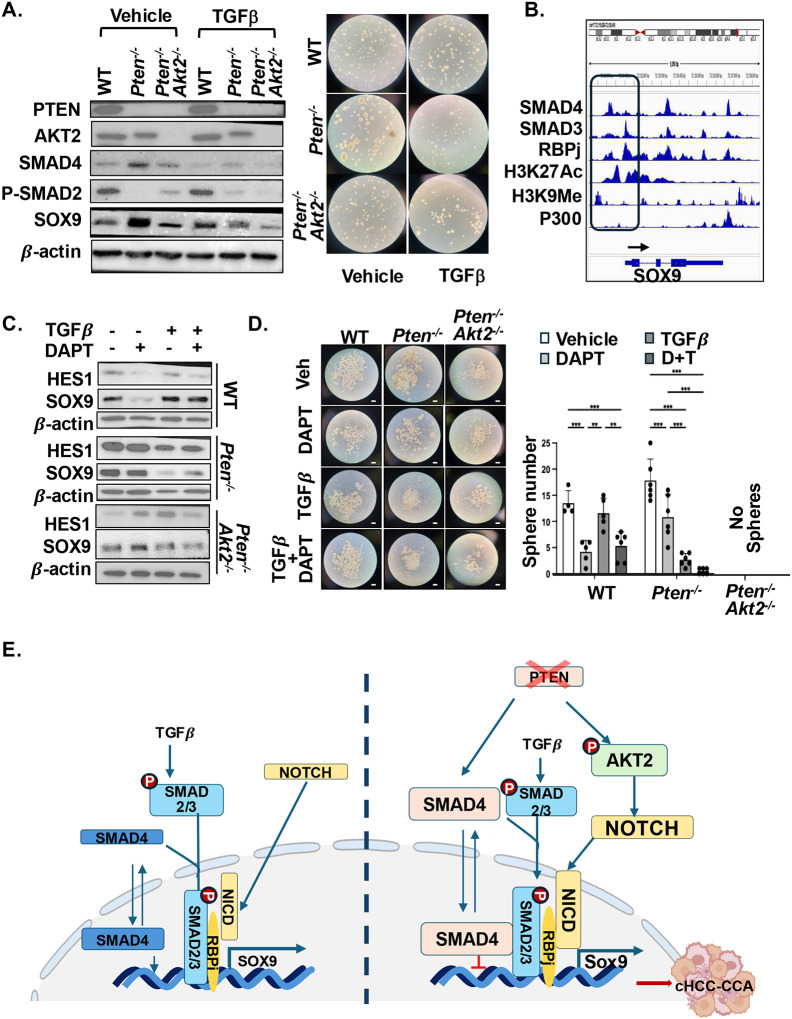
PTEN loss sensitizes liver tumor cells to TGFβ treatment. **(A)** Left, immunoblotting showing upregulation of SMAD4 with downregulation of SMAD2 phosphorylation in *Pten*^*−/−*^ cells. This is partially rescued by AKT2 loss (*Pten*^*−/−*^*; Akt2*^*−/−*^). TGFβ induces SOX9 in WT cells but inhibits it in cells lacking PTEN. AKT2 loss reverses the effect of PTEN loss. Right, representative image showing TGFβ treatment inhibits sphere formation in *Pten*^*−/−*^ cells but not WT cells and cells lacking AKT2 (*Pten*^*−/−*^*; Akt2*^*−/−*^). **(B)** Promoter analysis of SOX9 showing binding site for SMAD3, SMAD4 and RBPj where SMAD3 and RBPj bind to the same site, where H3K27Ac marks a transcriptional activation site. SMAD4 binds to a different site. A H3K9Me site is adjacent to the SMAD4 binding site. **(C)** Immunoblotting analysis of spheres formed in WT, *Pten*^*−/−*^ and *Pten*^*−/−*^*; Akt2*^*−/−*^ immortalized cells treated with TGFβ, DAPT or both. TGFβ inhibits SOX9 in *Pten*^*−/−*^ and *Pten*^*−/−*^*; Akt2*^*−/−*^ cells whereas DAPT inhibits SOX9 in WT cells. Blots are cropped for clarity. **(D)** Sphere formation in WT, *Pten*^*−/−*^ and *Pten*^*−/−*^*; Akt2*^*−/−*^ immortalized cells treated with TGFβ, DAPT or both (D+T). Left, representative images. Right, quantification of sphere numbers. No cell cluster in and *Pten*^*−/−*^*; Akt2*^*/-*^ cells reached the threshold of spheres.at day 3 of sphere culture. Scale bar 100 μm. n=6. Experiment repeated 3 times. ** p< 0.01, *** p< 0.001. **(E)** Proposed schematics for how PTEN-AKT2 signal regulates the response of LCSC to TGFβ through the regulation of SOX9.

Here, we found that TGFβ treatment differentially regulates SOX9 expression depending on PTEN status. Specifically, TGFβ induced SOX9 expression in WT cells but suppressed it in*Pten*^−/−^ cells (Fig. 8A), indicating that PTEN loss fundamentally alters the cellular response to TGFβ. Functionally, TGFβ treatment robustly and significantly reduced sphere formation in *Pten*^−/−^ cells, whereas WT cells were largely unresponsive. Notably, spheres formed by TGFβ-treated WT cells were modestly larger than untreated controls (Fig. 8A). In cells lacking both PTEN and AKT2, TGFβ continued to suppress SOX9 expression despite reduced SMAD4 protein levels; however, TGFβ treatment no longer significantly affected sphere number or size (Fig. 8A). These data indicate that signals downstream of TGFβ play a role in PTEN-dependent tumor transformation, whereas AKT2 loss uncouples TGFβ-mediated SOX9 repression from functional inhibition of stem-like growth.

To investigate the molecular basis by which TGFβ regulates SOX9 expression, we analyzed the SOX9 promoter using ENCODE datasets^20^ from HepG2 cells. This analysis revealed binding of both SMAD3 and SMAD4 within 1 kb of the SOX9 transcriptional start site (Fig. 8B), supporting direct regulation by TGFβ signaling. Notably, SMAD3 binds between two H3K27Ac peaks, characteristic of active chromatin, whereas SMAD4 occupies a distinct site positioned between H3K27Ac and H3K9me marks, which denote active and repressive chromatin states, respectively. This configuration is unique to the SOX9 promoter and differs from canonical TGFβ target genes such as *SERPINE1* and *CCN2*, where SMAD3 and SMAD4 co-occupy the same active regulatory regions (**Supplementary Fig. 7**). This distinct positioning of SMAD4 on the SOX9 promoter could be involved in converting an open chromatin to a closed chromatin configuration^42^, via recruitment of different transcriptional complexes by SMAD4^43^ in PTEN-deficient cells. Additionally, RBPj, the transcriptional effector of NOTCH signaling, co-localizes with SMAD3 at sites across TGFβ-responsive genes including Sox9, supporting crosstalk of the two signaling pathways.

We next examined the functional consequences of TGFβ and NOTCH pathway interactions by treating cells with TGFβ in combination with DAPT. As shown (Fig. 7A), PTEN loss renders cells less responsive to DAPT in sphere formation assays. Consistent with this, DAPT failed to reduce HES1 or SOX9 levels in *Pten*^−/−^ cells, whereas the same dose effectively suppressed both targets in WT cells (Fig. 8C). In contrast, TGFβ treatment robustly suppressed HES1 and SOX9 in *Pten*^−/−^ cells. These differential responses were mirrored functionally: WT cells were sensitive to DAPT but resistant to TGFβ-mediated inhibition of sphere formation, whereas *Pten*^−/−^ cells responded to TGFβ, with no additional effect from DAPT (Fig. 8D). Cells lacking both PTEN and AKT2 were similarly unresponsive to DAPT, regardless of TGFβ treatment.

Together, these findings support a model in which cells with intact PTEN rely on NOTCH signaling to maintain progenitor or cholangiocytic identity, with TGFβ reinforcing this program. In contrast, PTEN loss—via AKT2—potentiates NOTCH–SOX9 signaling to drive LCSC transformation, while simultaneously sensitizing cells to TGFβ-mediated repression, likely through altered SMAD4-dependent chromatin regulation (Fig. 8E). This TGFβ signaling switch does not require AKT2, despite AKT2-dependent regulation of SMAD4 protein levels, suggesting that SMAD4-mediated repression may prevent NICD-driven stemness programs when AKT2 is absent.

## Discussion

In this study, we report that PTEN, through AKT2, plays a crucial role in regulating the development of mixed lineage tumor phenotypes. We showed that 1) loss of PTEN in various parenchymal cells – albumin-positive, SOX9-positive, or mature hepatocytes- results in iCCA-HCC cancer development regardless of cell origin; 2) deletion of *Akt2* blocks the PTEN-regulated mixed-lineage cancer development; 3) SOX9, a NOTCH-induced transcriptional factor, regulates LCSC transformation downstream of PTEN/AKT2; and 4) TGFβ is required to block SOX9 and LCSC transformation in the absence of PTEN. Together, these data established the role of the PTEN/AKT2 signal in mixed lineage liver cancer development and delineated TGFβ regulated pathways that contribute to this development (Fig. 8E).

Primary liver cancer arises from either hepatocytic or biliary lineage cells, giving rise to HCC or iCCA, respectively. Although traditionally considered distinct entities, accumulating molecular and histological evidence supports a continuum of lineage plasticity across primary liver tumors, exemplified by the aggressive combined cHCC-CCA, which displays features of both lineages within the same lesion^44,45^. Using a lineage tracing approach, we established that the PTEN/AKT2 signal plays critical roles in the cHCC-CCA development. Without PTEN, cHCC-CCA develops irrespective of the cell type that deletion of *Pten* is targeted to, indicating that PTEN loss overrides lineage-specific constraints. Importantly, this process requires AKT2, identifying it as a critical oncogenic effector downstream of PTEN. AKT2 is the most abundantly expressed AKT isoform in the liver^29,30^. We have previously reported that germline AKT2 loss inhibits tumor development in the LiPten mice^14^. We showed that AKT2-regulated lipid biosynthesis produces a tumor microenvironment to promote the oncogenic events induced by PTEN loss^28,31^. However, AKTs were found to be dispensable for chemical-induced liver carcinogenesis though cell context differences appear to determine how each AKT isoforms function^30^. We established here that AKT2 indeed plays a role in hepatocyte-cholangiocyte cell fate regulated by PTEN. Whether this function depends on it being a metabolic kinase remains to be determined. Given that activation of AKT is an unequivocal consequence observed with PTEN loss of function^46^, our current work highlights a context-specific requirement for AKT2 in liver tumorigenesis and suggests that AKT isoform dependency may underlie divergent outcomes observed in other models of liver cancer. Future work is needed to directly compare the effects of each AKT isoform to decipher their unique cell-specific functions.

Molecularly, cHCC-CCA may originate from LCSCs capable of dual hepatocytic and cholangiocytic differentiation^44^, or through transdifferentiation of mature hepatocytes into cholangiocyte-like cells, a process often triggered by chronic injury or oncogenic signaling^47^. These lineage conversions are orchestrated by key developmental pathways—including aberrant activation of developmental signaling networks including NOTCH and WNT/β-CATENIN— which enable the emergence of tumors containing both hepatocytic and cholangiocytic components^47–49^. Here, we report that loss of PTEN induces the upregulation of NOTCH signal, concomitant with the induction of WNT/β-CATENIN that we characterized previously^19^, reinforcing a stemness-promoting transcriptional program. In addition to NOTCH and WNT signals, we identified TGFβ as a key context-dependent regulator of lineage fate decisions downstream of PTEN loss. While TGFβ can induce the expression of SOX9 during biliary differentiation, we show that sustained upregulation of SMAD4 in PTEN-deficient cells permits TGFβ to repress SOX9 and inhibit SOX9driven LCSC transformation. This observation is similar to those observed in liver LCSCs with sustained TGFβR II activity^50,51^. These results suggest that TGFβ and NOTCH converge onto SOX9 to control LCSC and that PTEN loss fundamentally alters cellular responsiveness, and sensitizes tumor cells to TGFβ mediated repression rather than NOTCH inhibition. Mechanistically, chronic upregulation of SMAD4 in the *Pten*^*−/−*^ cells may promote recruitment of repressive SMAD4 complexes to the SOX9 promoter, facilitating a shift from an open to a closed chromatin configuration and converting TGFβ signaling from a pro–stemness to an anti–stemness program (Fig. 8E). Collectively, these findings define PTEN–AKT2 signaling as a critical determinant of lineage plasticity in liver cancer and reveal a previously unrecognized mechanism by which developmental signaling pathways are rewired to drive mixed-lineage tumor development.

In summary, our study demonstrates that PTEN–PI3K–AKT2 signaling is a key regulator of hepatocyte–cholangiocyte fate determination and a central driver of cHCC–CCA development. We identify AKT2 as the critical AKT isoform mediating this mixed-lineage tumor phenotype, providing a mechanistic rationale for isoform-specific targeting of AKT signaling in liver cancer. Furthermore, we uncover a PTEN-dependent crosstalk between TGFβ and NOTCH signaling that converges on SOX9 to regulate tumor lineage plasticity and show that PTEN loss sensitizes tumor cells to TGFβ-mediated repression. Together, these findings define a molecular framework linking tumor suppressor loss, developmental signaling, and lineage plasticity, with important implications for therapeutic strategies in aggressive liver cancers.

## Supplementary Material

Supplementary Files

This is a list of supplementary files associated with this preprint. Click to download.

- SupplementaryMaterials.pdf

## Data Availability

The data generated in this study are available within the article and its supplementary data files. The data regenerated from the database are available at the public data depository as indicated in the results section.
